# 5-[(1*H*-Benzimidazol-1-yl)meth­yl]benzene-1,3-dicarb­oxy­lic acid

**DOI:** 10.1107/S1600536811047416

**Published:** 2011-11-12

**Authors:** Xiao-Chun Cheng

**Affiliations:** a Faculty of Life Science and Chemical Engineering, Huaiyin Institute of Technology, Huaian 223003, People’s Republic of China

## Abstract

Crystals of the title compound, C_16_H_12_N_2_O_4_, were obtained accidentally from a hydro­thermal reaction of 5-[(1*H*-benzimidazol-1-yl)meth­yl]isophthalic acid with manganese bromide in the presence of *N*,*N*′-dimethyl­formamide. In the title mol­ecule, the benzimidazole ring system is almost planar, with a maximum deviation from the mean plane of 0.010 (2) Å. The benzimidazole and central benzene rings are inclined at a dihedral angle of 71.7 (6)°. The crystal structure is stabilized by O—H⋯N and O—H⋯O hydrogen bonds.

## Related literature

For background information on the title compound, see: Das & Bharadwaj (2009 ▶). For a related structure, see: Kuai & Cheng (2011 ▶).
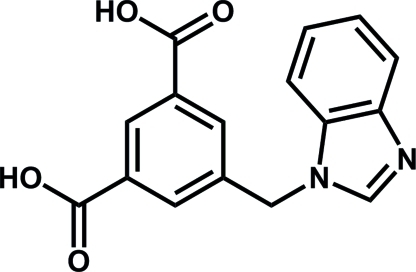


## Experimental

### 

#### Crystal data

C_16_H_12_N_2_O_4_
*M* *_r_* = 296.28
Triclinic, 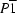

*a* = 7.7159 (11) Å
*b* = 8.4559 (12) Å
*c* = 10.9742 (15) Å
α = 97.286 (2)°
β = 104.928 (2)°
γ = 98.029 (2)°
*V* = 675.07 (16) Å^3^
*Z* = 2
Mo *K*α radiation
μ = 0.11 mm^−1^
*T* = 293 K
0.20 × 0.20 × 0.20 mm

#### Data collection

Bruker SMART APEXII CCD diffractometer
Absorption correction: multi-scan (*SADABS*; Sheldrick, 1996 ▶) *T*_min_ = 0.979, *T*_max_ = 0.979
3620 measured reflections
2499 independent reflections
1401 reflections with *I* > 2σ(*I*)
*R*_int_ = 0.024

#### Refinement

*R*[*F* ^2^ > 2σ(*F* ^2^)] = 0.042
*wR*(*F* ^2^) = 0.098
*S* = 0.84
2499 reflections
199 parameters
H-atom parameters constrained
Δρ_max_ = 0.18 e Å^−3^
Δρ_min_ = −0.17 e Å^−3^

### 

Data collection: *APEX2* (Bruker, 2008 ▶); cell refinement: *SAINT* (Bruker, 2008 ▶); data reduction: *SAINT*; program(s) used to solve structure: *SHELXS97* (Sheldrick, 2008 ▶); program(s) used to refine structure: *SHELXL97* (Sheldrick, 2008 ▶); molecular graphics: *DIAMOND* (Brandenburg, 2000 ▶); software used to prepare material for publication: *SHELXTL* (Sheldrick, 2008 ▶).

## Supplementary Material

Crystal structure: contains datablock(s) I, global. DOI: 10.1107/S1600536811047416/pv2480sup1.cif

Structure factors: contains datablock(s) I. DOI: 10.1107/S1600536811047416/pv2480Isup2.hkl

Supplementary material file. DOI: 10.1107/S1600536811047416/pv2480Isup3.cdx

Supplementary material file. DOI: 10.1107/S1600536811047416/pv2480Isup4.cml

Additional supplementary materials:  crystallographic information; 3D view; checkCIF report

## Figures and Tables

**Table 1:** Hydrogen-bond geometry (Å, °)

*D*—H⋯*A*	*D*—H	H⋯*A*	*D*⋯*A*	*D*—H⋯*A*
O3—H12⋯O2^i^	0.82	1.84	2.574 (2)	147
O1—H11⋯N2^ii^	0.82	1.76	2.553 (2)	162

Symmetry codes: (i) 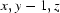; (ii) 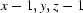.

**Fig. 1.**
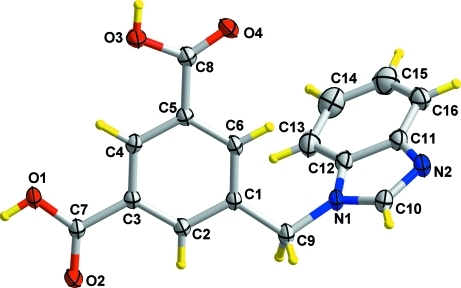
: The crystal structure of the title compound showing 30% probability displacement ellipsoids.

## supplementary crystallographic information

### Comment

The title compound is regarded as an excellent candidate for building block in molecular self-assembly engineering due to its variable conformation and coordination modes (Das & Bharadwaj, 2009). During preparation of coordination polymers, we accidentally obtained single crystals of the title compound by the hydrothermal reaction at 393 K of 5-((1*H*-benzo[*d*]imidazol-1-yl)methyl)isophthalic acid with manganese bromide in the presence of *N*,*N*'-dimethylformamide.

Although crystallized from an alkaline solution, the title compound retained the carboxylic groups in the crystal structure (Fig. 1). The benzimidazolyl ring and the central benzene ring are inclined at a dihedral angle of 71.7 (6) °. In the crystal structure, there exist O—H···N and O—H···O hydrogen bonds (Table 1). The carboxylate groups and the N atom in the benzimidazolyl group as donor or acceptor play very important role in the formation of these hydrogen bonds.

### Experimental

A mixture of MnBr_2_ (21.5 mg, 0.1 mmol), 5-((1*H*-benzo[*d*]imidazol-1-yl)methyl)isophthalic acid (29.6 mg, 0.1 mmol) and 2 ml *N*,*N*'-dimethylformamide (DMF) in 10 ml H_2_O was sealed in a 16 ml Teflon-lined stainless steel container and heated to 393 K for 3 days. After cooling the stain-less steel container to room temperature, colorless block crystals of the title compound were obtained.

### Refinement

All hydrogen atoms were included at geometrically idealized positions and constrained to ride on their parent atoms, with C—H = 0.93 or 0.97, O—H = 0.82 Å and *U*_iso_(H) = 1.2*U*_eq_(C/O).

### Crystal data

**Table:**

C_16_H_12_N_2_O_4_	*Z* = 2
*M**_r_* = 296.28	*F*(000) = 308
Triclinic, *P*1	*D*_x_ = 1.458 Mg m^−^^3^
Hall symbol: -P 1	Mo *K*α radiation, λ = 0.71073 Å
*a* = 7.7159 (11) Å	Cell parameters from 796 reflections
*b* = 8.4559 (12) Å	θ = 2.8–25.0°
*c* = 10.9742 (15) Å	µ = 0.11 mm^−^^1^
α = 97.286 (2)°	*T* = 293 K
β = 104.928 (2)°	Block, colorless
γ = 98.029 (2)°	0.20 × 0.20 × 0.20 mm
*V* = 675.07 (16) Å^3^	

### Data collection

**Table:**

Bruker SMART APEXII CCD diffractometer	2499 independent reflections
Radiation source: fine-focus sealed tube	1401 reflections with *I* > 2σ(*I*)
graphite	*R*_int_ = 0.024
φ and ω scans	θ_max_ = 25.6°, θ_min_ = 2.0°
Absorption correction: multi-scan (*SADABS*; Sheldrick, 1996)	*h* = −8→9
*T*_min_ = 0.979, *T*_max_ = 0.979	*k* = −10→9
3620 measured reflections	*l* = −11→13

### Refinement

**Table:**

Refinement on *F*^2^	Primary atom site location: structure-invariant direct methods
Least-squares matrix: full	Secondary atom site location: difference Fourier map
*R*[*F*^2^ > 2σ(*F*^2^)] = 0.042	Hydrogen site location: inferred from neighbouring sites
*wR*(*F*^2^) = 0.098	H-atom parameters constrained
*S* = 0.84	*w* = 1/[σ^2^(*F*_o_^2^) + (0.0392*P*)^2^] where *P* = (*F*_o_^2^ + 2*F*_c_^2^)/3
2499 reflections	(Δ/σ)_max_ < 0.001
199 parameters	Δρ_max_ = 0.18 e Å^−^^3^
0 restraints	Δρ_min_ = −0.17 e Å^−^^3^

### Special details

**Table:**

Geometry. All e.s.d.'s (except the e.s.d. in the dihedral angle between two l.s. planes) are estimated using the full covariance matrix. The cell e.s.d.'s are taken into account individually in the estimation of e.s.d.'s in distances, angles and torsion angles; correlations between e.s.d.'s in cell parameters are only used when they are defined by crystal symmetry. An approximate (isotropic) treatment of cell e.s.d.'s is used for estimating e.s.d.'s involving l.s. planes.
Refinement. Refinement of *F*^2^ against ALL reflections. The weighted *R*-factor *wR* and goodness of fit *S* are based on *F*^2^, conventional *R*-factors *R* are based on *F*, with *F* set to zero for negative *F*^2^. The threshold expression of *F*^2^ > σ(*F*^2^) is used only for calculating *R*-factors(gt) *etc*. and is not relevant to the choice of reflections for refinement. *R*-factors based on *F*^2^ are statistically about twice as large as those based on *F*, and *R*- factors based on ALL data will be even larger.

### Fractional atomic coordinates and isotropic or equivalent isotropic displacement parameters (Å^2^)

**Table:**

	*x*	*y*	*z*	*U*_iso_*/*U*_eq_	
C1	0.8649 (3)	0.3096 (2)	0.61209 (17)	0.0400 (5)	
C2	0.7615 (3)	0.3686 (2)	0.50979 (18)	0.0426 (5)	
H1	0.7649	0.4796	0.5144	0.051*	
C3	0.6533 (3)	0.2632 (2)	0.40097 (17)	0.0378 (5)	
C4	0.6482 (3)	0.0979 (2)	0.39490 (17)	0.0386 (5)	
H2	0.5757	0.0268	0.3222	0.046*	
C5	0.7503 (3)	0.0376 (2)	0.49609 (17)	0.0368 (5)	
C6	0.8591 (3)	0.1451 (2)	0.60419 (18)	0.0414 (5)	
H3	0.9289	0.1052	0.6720	0.050*	
C7	0.5439 (3)	0.3280 (3)	0.29147 (18)	0.0442 (6)	
C8	0.7429 (3)	−0.1397 (3)	0.4906 (2)	0.0443 (6)	
C9	0.9807 (3)	0.4336 (3)	0.72598 (18)	0.0572 (7)	
H5	1.0883	0.4833	0.7053	0.069*	
H4	0.9121	0.5180	0.7417	0.069*	
C10	1.2110 (3)	0.3646 (3)	0.9087 (2)	0.0513 (6)	
H6	1.3127	0.4067	0.8847	0.062*	
C11	1.0403 (3)	0.2516 (2)	1.01338 (19)	0.0470 (6)	
C12	0.9243 (3)	0.2953 (3)	0.9078 (2)	0.0462 (6)	
C13	0.7369 (3)	0.2658 (3)	0.8840 (2)	0.0618 (7)	
H7	0.6604	0.2964	0.8140	0.074*	
C14	0.6703 (4)	0.1887 (3)	0.9698 (3)	0.0737 (8)	
H8	0.5448	0.1652	0.9568	0.088*	
C15	0.7851 (4)	0.1444 (3)	1.0760 (2)	0.0706 (8)	
H9	0.7340	0.0933	1.1320	0.085*	
C16	0.9705 (4)	0.1743 (3)	1.0997 (2)	0.0580 (7)	
H10	1.0465	0.1443	1.1702	0.070*	
N1	1.0380 (2)	0.3660 (2)	0.84266 (15)	0.0468 (5)	
N2	1.2195 (2)	0.2965 (2)	1.01142 (15)	0.0500 (5)	
O1	0.44624 (19)	0.21783 (16)	0.19787 (12)	0.0559 (5)	
H11	0.3897	0.2606	0.1410	0.067*	
O2	0.5500 (2)	0.47226 (18)	0.29074 (14)	0.0763 (6)	
O3	0.6485 (2)	−0.22162 (17)	0.37611 (13)	0.0693 (5)	
H12	0.6465	−0.3189	0.3759	0.083*	
O4	0.8127 (2)	−0.20331 (17)	0.57734 (14)	0.0627 (5)	

### Atomic displacement parameters (Å^2^)

**Table:**

	*U*^11^	*U*^22^	*U*^33^	*U*^12^	*U*^13^	*U*^23^
C1	0.0523 (13)	0.0322 (12)	0.0251 (11)	0.0059 (10)	−0.0061 (10)	0.0038 (9)
C2	0.0582 (14)	0.0295 (12)	0.0301 (11)	0.0073 (10)	−0.0053 (10)	0.0060 (9)
C3	0.0472 (12)	0.0316 (12)	0.0269 (11)	0.0057 (10)	−0.0024 (9)	0.0049 (9)
C4	0.0488 (12)	0.0319 (12)	0.0260 (11)	0.0045 (10)	−0.0026 (9)	0.0024 (9)
C5	0.0457 (12)	0.0308 (11)	0.0285 (11)	0.0074 (10)	0.0006 (10)	0.0056 (9)
C6	0.0524 (13)	0.0375 (13)	0.0277 (11)	0.0109 (10)	−0.0040 (10)	0.0103 (9)
C7	0.0560 (14)	0.0338 (13)	0.0306 (12)	0.0073 (11)	−0.0087 (10)	0.0049 (10)
C8	0.0558 (14)	0.0362 (13)	0.0347 (12)	0.0078 (11)	0.0015 (11)	0.0072 (10)
C9	0.0806 (16)	0.0403 (14)	0.0310 (12)	0.0045 (12)	−0.0160 (12)	0.0063 (10)
C10	0.0565 (14)	0.0437 (14)	0.0379 (13)	0.0015 (11)	−0.0093 (11)	0.0046 (11)
C11	0.0629 (16)	0.0365 (13)	0.0314 (12)	0.0094 (11)	−0.0025 (11)	0.0016 (10)
C12	0.0590 (15)	0.0357 (13)	0.0322 (12)	0.0090 (11)	−0.0042 (11)	−0.0013 (10)
C13	0.0595 (17)	0.0625 (18)	0.0514 (16)	0.0143 (13)	−0.0037 (13)	0.0015 (13)
C14	0.0649 (17)	0.080 (2)	0.0678 (19)	0.0068 (15)	0.0142 (16)	−0.0015 (16)
C15	0.084 (2)	0.0659 (19)	0.0587 (18)	0.0081 (16)	0.0218 (16)	0.0031 (14)
C16	0.0863 (19)	0.0445 (15)	0.0366 (14)	0.0116 (14)	0.0055 (14)	0.0072 (11)
N1	0.0584 (12)	0.0393 (11)	0.0279 (10)	0.0077 (9)	−0.0126 (9)	0.0047 (8)
N2	0.0597 (12)	0.0471 (12)	0.0320 (10)	0.0092 (10)	−0.0076 (9)	0.0081 (9)
O1	0.0717 (10)	0.0413 (9)	0.0345 (8)	0.0105 (8)	−0.0204 (8)	0.0056 (7)
O2	0.1136 (14)	0.0305 (9)	0.0515 (10)	0.0101 (9)	−0.0338 (9)	0.0070 (7)
O3	0.1078 (13)	0.0281 (9)	0.0467 (10)	0.0108 (9)	−0.0202 (9)	0.0025 (7)
O4	0.0939 (12)	0.0384 (9)	0.0431 (9)	0.0145 (9)	−0.0078 (9)	0.0142 (7)

### Geometric parameters (Å, °)

**Table:**

C1—C6	1.377 (3)	C9—H4	0.9700
C1—C2	1.392 (2)	C10—N2	1.320 (3)
C1—C9	1.513 (2)	C10—N1	1.347 (2)
C2—C3	1.387 (2)	C10—H6	0.9300
C2—H1	0.9300	C11—N2	1.388 (3)
C3—C4	1.386 (2)	C11—C16	1.391 (3)
C3—C7	1.493 (3)	C11—C12	1.395 (3)
C4—C5	1.385 (2)	C12—C13	1.382 (3)
C4—H2	0.9300	C12—N1	1.387 (3)
C5—C6	1.392 (2)	C13—C14	1.374 (4)
C5—C8	1.485 (3)	C13—H7	0.9300
C6—H3	0.9300	C14—C15	1.398 (3)
C7—O2	1.215 (2)	C14—H8	0.9300
C7—O1	1.286 (2)	C15—C16	1.367 (3)
C8—O4	1.197 (2)	C15—H9	0.9300
C8—O3	1.325 (2)	C16—H10	0.9300
C9—N1	1.458 (3)	O1—H11	0.8200
C9—H5	0.9700	O3—H12	0.8200
			
C6—C1—C2	119.24 (17)	H5—C9—H4	107.7
C6—C1—C9	123.83 (17)	N2—C10—N1	112.3 (2)
C2—C1—C9	116.92 (18)	N2—C10—H6	123.9
C3—C2—C1	120.61 (19)	N1—C10—H6	123.9
C3—C2—H1	119.7	N2—C11—C16	130.2 (2)
C1—C2—H1	119.7	N2—C11—C12	109.0 (2)
C4—C3—C2	119.40 (18)	C16—C11—C12	120.8 (2)
C4—C3—C7	120.48 (17)	C13—C12—N1	132.3 (2)
C2—C3—C7	120.12 (18)	C13—C12—C11	122.4 (2)
C5—C4—C3	120.58 (17)	N1—C12—C11	105.34 (19)
C5—C4—H2	119.7	C14—C13—C12	116.1 (2)
C3—C4—H2	119.7	C14—C13—H7	122.0
C4—C5—C6	119.27 (18)	C12—C13—H7	122.0
C4—C5—C8	120.67 (17)	C13—C14—C15	122.1 (3)
C6—C5—C8	120.06 (17)	C13—C14—H8	119.0
C1—C6—C5	120.90 (17)	C15—C14—H8	119.0
C1—C6—H3	119.6	C16—C15—C14	121.7 (3)
C5—C6—H3	119.6	C16—C15—H9	119.1
O2—C7—O1	123.42 (18)	C14—C15—H9	119.1
O2—C7—C3	122.59 (18)	C15—C16—C11	116.9 (2)
O1—C7—C3	113.98 (18)	C15—C16—H10	121.5
O4—C8—O3	123.1 (2)	C11—C16—H10	121.5
O4—C8—C5	125.28 (19)	C10—N1—C12	107.49 (17)
O3—C8—C5	111.58 (18)	C10—N1—C9	126.3 (2)
N1—C9—C1	113.68 (18)	C12—N1—C9	126.15 (19)
N1—C9—H5	108.8	C10—N2—C11	105.86 (18)
C1—C9—H5	108.8	C7—O1—H11	109.5
N1—C9—H4	108.8	C8—O3—H12	109.5
C1—C9—H4	108.8		
			
C6—C1—C2—C3	0.1 (3)	N2—C11—C12—C13	179.95 (18)
C9—C1—C2—C3	179.18 (19)	C16—C11—C12—C13	0.5 (3)
C1—C2—C3—C4	0.2 (3)	N2—C11—C12—N1	0.4 (2)
C1—C2—C3—C7	−179.6 (2)	C16—C11—C12—N1	−179.05 (19)
C2—C3—C4—C5	−0.1 (3)	N1—C12—C13—C14	178.6 (2)
C7—C3—C4—C5	179.7 (2)	C11—C12—C13—C14	−0.8 (3)
C3—C4—C5—C6	−0.3 (3)	C12—C13—C14—C15	0.8 (4)
C3—C4—C5—C8	179.06 (19)	C13—C14—C15—C16	−0.6 (4)
C2—C1—C6—C5	−0.6 (3)	C14—C15—C16—C11	0.3 (4)
C9—C1—C6—C5	−179.6 (2)	N2—C11—C16—C15	−179.5 (2)
C4—C5—C6—C1	0.7 (3)	C12—C11—C16—C15	−0.2 (3)
C8—C5—C6—C1	−178.7 (2)	N2—C10—N1—C12	0.8 (2)
C4—C3—C7—O2	−177.0 (2)	N2—C10—N1—C9	179.20 (18)
C2—C3—C7—O2	2.9 (3)	C13—C12—N1—C10	179.8 (2)
C4—C3—C7—O1	1.7 (3)	C11—C12—N1—C10	−0.7 (2)
C2—C3—C7—O1	−178.42 (18)	C13—C12—N1—C9	1.4 (4)
C4—C5—C8—O4	−172.8 (2)	C11—C12—N1—C9	−179.11 (18)
C6—C5—C8—O4	6.6 (3)	C1—C9—N1—C10	118.4 (2)
C4—C5—C8—O3	7.3 (3)	C1—C9—N1—C12	−63.5 (3)
C6—C5—C8—O3	−173.30 (18)	N1—C10—N2—C11	−0.5 (2)
C6—C1—C9—N1	−19.5 (3)	C16—C11—N2—C10	179.4 (2)
C2—C1—C9—N1	161.5 (2)	C12—C11—N2—C10	0.1 (2)

### Hydrogen-bond geometry (Å, °)

**Table:**

*D*—H···*A*	*D*—H	H···*A*	*D*···*A*	*D*—H···*A*
O3—H12···O2^i^	0.82	1.84	2.574 (2)	147.
O1—H11···N2^ii^	0.82	1.76	2.553 (2)	162.

Symmetry codes: (i) *x*, *y*−1, *z*; (ii) *x*−1, *y*, *z*−1.
